# A Mini Review of the Preparation and Photocatalytic Properties of Two-Dimensional Materials

**DOI:** 10.3389/fchem.2020.582146

**Published:** 2020-12-09

**Authors:** Shuhua Hao, Xinpei Zhao, Qiyang Cheng, Yupeng Xing, Wenxuan Ma, Xiaoke Wang, Gang Zhao, Xijin Xu

**Affiliations:** ^1^Laboratory of Functional Micro and Nano Materials and Devices, School of Physics and Technology, University of Jinan, Jinan, China; ^2^Department of Chemical Engineering and Safety, Binzhou University, Binzhou, China

**Keywords:** two-dimensional materials, preparation method, photocatalytic properties, catalytic effect, hydrogen production

## Abstract

The successful preparation and application of graphene shows that it is feasible for the materials with a thickness of a single atom or few atomic layers to exist stably in nature. These materials can exhibit unusual physical and chemical properties due to their special dimension effects. At present, researchers have made great achievements in the preparation, characterization, modification, and theoretical research of 2D materials. Because the structure of 2D materials is often similar, it has a certain degree of qualitative versatility. Besides, 2D materials often carry good catalytic performance on account of their more active sites and adjustable harmonic electronic structure. In this review, taking 2D materials as examples [graphene, boron nitride (h-BN), transition metal sulfide and so on], we review the crystal structure and preparation methods of these materials in recent years, focus on their photocatalyst properties (carbon dioxide reduction and hydrogen production), and discuss their applications and development prospects in the future.

## Introduction

By physically or chemically weakening the intermolecular forces, many layered materials can be prepared as monolayer or less layered materials, which tend to take on a nanoscale appearance in one direction and are called 2D materials. As the dimension changes, the properties of these 2D materials are often changed. A typical example is graphene, which has been widely reported due to its unique properties, including spectral absorption, high specific surface area, high young's modulus, high carrier mobility, and molecular barrier.

After the discovery of the excellent properties of graphene, more researchers focused their research on 2D materials, and subsequently discovered more 2D materials (Novoselov et al., 2004). Jacoby made a series of summaries of 2D materials: according to the periodic table, 2D materials can be divided into five categories, including transition metal carbide and nitride (MXenes), single element alkene, organic materials, nitride and transition metal dihalides (Kang et al., 2016). Many studies have shown that the above mentioned 2D materials have a broad application prospect. In all the researches on the properties and applications of 2D materials, catalytic performance is always a particularly important core direction. Under the mediation of its structure and electron specificity, 2D materials have good activities in photocatalytic carbon dioxide reduction and hydrogen production, and the properties of 2D materials are related to their preparation method, crystal structure, and doping type (Guardia et al., 2014; Cai and Feng, 2016; Sun et al., 2018).

In this review, we will briefly describe the crystal structure and preparation methods of 2D materials [graphene, hexagonal boron nitride (h-BN), transition metal disulfide and so on], and their applications in the photocatalytic field, such as hydrogen production and carbon dioxide reduction. Then, we focus on the photocatalytic properties and performance regulation of the above-mentioned materials, and summarize all aspects of the 2D materials, and discuss the potential of 2D materials in future scientific development.

## Several Methods of Exfoliating 2D Materials

**Figure 1:**
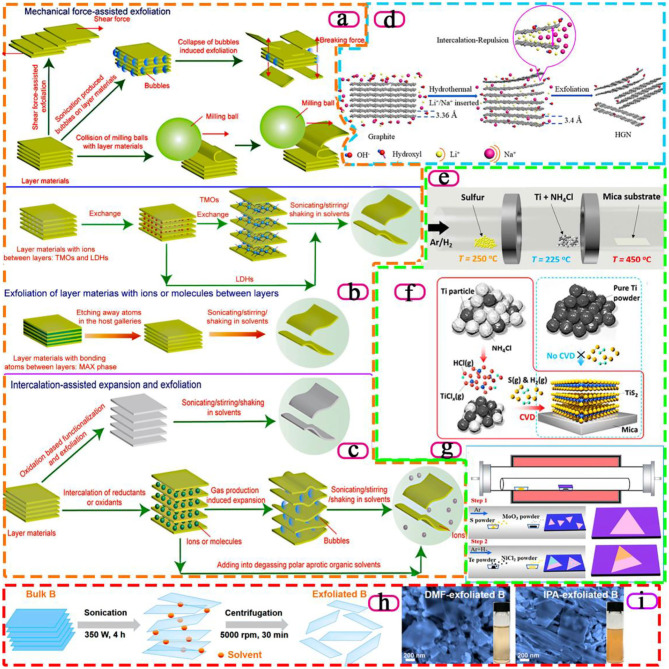
Schematics of different methods to prepare 2D materials by exfoliating layer materials. **(a)** Mechanical force-assisted exfoliation. **(b)** Exfoliation of layer materials with ions or molecules between layers. **(c)** Intercalation-assisted expansion and exfoliation, (Cai et al., 2018). Copyright 2018, Royal Society of Chemistry. **(d)** Schematic illustration of exfoliating graphite by intercalating graphite with alkali-metal ions (Cheng et al., 2019). Copyright 2019, American Chemical Society. **(e)** Experimental setup for the CVD growth of TiS_2_ nanosheets on mica substrates. **(f)** Schematic illustration of the CVD growth mechanism with NH_4_Cl promoters (Gao et al., 2018). Copyright 2018, American Chemical Society. **(g)** Schematic illustrating the two-step CVD growth of NiTe_2_/MoS_2_ heterostructure (Zhai et al., 2020). Copyright 2020, American Chemical Society. **(h)** Schematic illustration of the sonication-assisted liquid-phase exfoliation process. **(i)** B sheets obtained by tip-sonication in DMF and IPA, respectively (Li et al., 2018). Copyright 2018, American Chemical Society.

Since Novoselov et al. successfully exfoliated graphene in 2004, 2D materials have attracted wide attention due to their unique physical and chemical properties, and there is an increasing demand for them year by year (Novoselov et al., 2004). However, 2D material is still a new material, and the preparation method needs to be further improved. The common preparation process is often accompanied by some shortcomings, such as too low efficiency, product impurity, and even some risks, limiting the research and application of 2D material properties. At present, the more mature method is mainly mechanical exfoliation. That is, a variety of mechanical forces are used to resist the van der Waals force between layered materials, which generally can obtain the required form of materials, but the waste of raw materials is also a serious issue. In addition, in many studies, the redox method is often discussed concerning the preparation of 2D materials, because of the low energy consumption and high yield of reaction products in the reaction process. Although the method has a wide range of applications, it still has some shortcomings that are difficult to solve. For example, the spatial structure of 2D materials will be damaged in the process of redox reaction, which will affect the physical and chemical properties of 2D materials. At present, although the preparation of 2D materials by chemical vapor deposition method is a very common and mature technology, this method has certain risks, and it is accompanied by high cost, and low yield and doping amount (Novoselov et al., 2012). In addition to the methods mentioned above, there are many other methods for preparing 2D materials, most of which can only be used to prepare and exfoliate one or two 2D materials, and are often not universal. Cai et al. illustrated the process of various 2D material preparation methodologies with graphic pictures, including intercalation-assisted expansion and exfoliation, Mechanical force-assisted exfoliation and Exfoliation of layer materials with ions or molecules between layers (Cai et al., 2018), as shown in Figures 1a-c. In the following, we will summarize the research progress of these aspects and comment on the influence of these methods on the catalytic effect of 2D materials.

### Mechanical Exfoliation Method

Compared with other 2D material preparation methods, mechanical force-assisted exfoliation has such advantages as wide application, high yield, and high purity. Because this method usually does not require the addition of other substances other than auxiliary substances to increase mechanical force, impurities are rarely introduced into the process. The principle of the mechanical stripping method is to make use of various mechanical forces to offset the van der Waals forces between layers. The most typical examples of the preparation of graphene by mechanical stripping are Novoselov et al. (2004), two Nobel Prize winners in physics, who successfully used the adhesive force of tape to offset the van der Waals force between the layers of the material.

In some graphene grinding processes, special properties of auxillaries are often used to process 2D materials in order to improve the exfoliation speed and prevent the structure of 2D materials from being damaged. For example, Zhao et al. used the auxiliary effect of dimethylformamide dispersion medium to grind and centrifuge the original material with a ball mill (Zhao et al., 2010). It is worth mentioning that, compared with the original materials with a thickness of 30 ~ 80 nm, suspension of graphene was prepared by ultrasonic expansion of the original material in organic solvent, and the graphene prepared after centrifugation has a very small thickness of 0.8 ~ 1.8 nm. The thickness, which proved that the above method is simple to operate and has high yield, which can be used as a method for large-scale preparation of graphene, before and after the experiment indicated that the graphene obtained was one or more layers. Thus, this method is simple to operate and has high yield, which can be used as a method for large-scale preparation of graphene. Wu et al. propose a novel method to exfoliate hexagonal boron nitride (h-BN) powders to produce boron nitride nanosheets by applying pure shear ball milling (Wu et al., 2019). Applying a vertical load to the milling balls could alter their pattern of motion, which also increased their average tangential force and number of contacts, resulting in higher exfoliation and less impact action on the bulk h-BN powders.

Simple mechanical ultrasound can no longer efficiently exfoliate layered materials to meet the requirements of preparing 2D materials, which leads to the exploration of new mechanical ultrasonic methods. Zhao et al. search for all kinds of ultrasound machines and ultimately design an ultrasonic instrument with magnetic stirring, which is a high-efficiency exfoliation method to obtain abundant and superior graphene, h-BN, MoS_2_, and WS_2_ nanosheets in aqueous solution (Zhao et al., 2016). In addition, Aparna et al. used the auxiliary effect of methanol solution of 1-pyridine formic acid, and this auxiliary effect can be applied to other 2D materials besides graphene (Aparna et al., 2013). Ji et al. select eight representative 2D materials (graphite, h-BN, MoS_2_, WS_2_, black phosphorus, metalorganic framework Mn(C_6_H_8_O_4_) (H_2_O) and two kinds of layered coordination polymer) with diverse inter- and intra-layer bonding interactions for the study, proposing to use the ratio between the in-plane and out-of-plane elastic modulus (E) as a universal index, A_In/Out_ (= E_In−plane_/E_Out−of−plane_), to quantify the ease level of a 2D material's mechanical exfoliation (Ji et al., 2018).

### Intercalation-Assisted Expansion and Exfoliation

There are also some unusual ways to prepare 2D materials, such as intercalation-assisted expansion and exfoliation, in which small molecules, non-covalently bonded molecules or polymers are inserted into a layered material and the material is stripped by reducing the van der Waals force of the layered layer. Since the invention of graphene in 2004, researchers have also tried the method of an ionic interlayer to prepare 2D materials. A “Chemical Weathering” exfoliation of atom-thick transition metal dichalcogenides is proposed by Zhao et al. to efficiently exfoliate MoS_2_ and WS_2_ nanosheets (Zhao et al., 2015) As a result of the high chemical potentials of Na^+^ and OH^−^ in the solution, the infiltration of Na^+^ and OH^−^ into the interlayer space of MoS_2_ or WS_2_ crystal continuously occurs and leads to the accumulation of NaOH in the raw materials. When the concentration of NaOH in MoS_2_ or WS_2_ crystal exceeds the critical value (≈7.6%), the raw materials are eventually exfoliated into ultrathin pieces. As shown in Figure 1d, Cheng et al. develop Li^+^/Na^+^ co-intercalated exfoliation for graphene nanosheets by a hydrothermal method without the participation of any organic solvents (Cheng et al., 2019). The graphite is exfoliated into graphene nanosheets by intercalating graphite with different ratios of alkali-metal ions (Li^+^, Na^+^). The insertion of Li^+^ and Na^+^ expands the distance between graphene layers, which weakens the van der Waals force and effectively exfoliates graphene. Moreover, the thickness of the as-prepared graphene nanosheets was 2.65–2.78 nm.

The remarkable advantages of the Redox method are high production efficiency and low resource consumption. However, due to the large number of oxidant reducing agents used in the Redox process, environmental pollution is often caused in the preparation process, and defects may occur in the Redox process of 2D materials, resulting in performance differences of 2D materials. In addition, some 2D materials are difficult to prepare using the Redox method due to their inherent chemical properties.

The Hummers method is also a commonly used method for preparing 2D materials (Hummers and Offeman, 1958). After the reaction of graphite powder with potassium permanganate in concentrated sulfuric acid, graphene oxide flakes are obtained after stripping. The success of exfoliation is usually achieved by the action of functional groups such as C=O double bond and hydroxyl group produced by chemical reactions between layered structures (Georgakilas et al., 2012). Hou et al. studied the influence of the dosage of concentrated sulfuric acid (H_2_SO_4_) and potassium permanganate (KMnO_4_) as well as the particle size of graphite raw material on the preparation process and performance of graphene sheet (Hou et al., 2020). The increase of oxidation degree of graphite material is beneficial to the escape of graphite oxide (GO) and the increase of concentration of concentrated sulfuric acid and potassium permanganate can improve the oxidation degree. Notably, sodium nitrate and sulfuric acid will have a synergistic effect on the preparation of 2D materials.

In the study of preparation of MXene, Cheng et al. found that MXene containing precious metal nanoparticles can undergo redox reaction (Cheng et al., 2020). In the reaction, the rare metal cation can absorb the electrons provided by MXene, so that MXene can be used as the reaction reducing agent without adding exogenous reducing agent, which reduces the pollution caused by introducing an exogenous reducing agent during the experiment. Although the above experiments did not solve the relationship between the preparation process of 2D materials and their physical/chemical properties, this study provided us with a new research idea.

### Chemical Vapor Deposition

A common bottom-up method for preparing 2D films of materials is chemical vapor deposition (CVD), in which solid sediments are formed by chemical reactions to form films on the substrate surface, especially in the preparation of transition metal disulfide films. However, the preparation of 2D materials by vapor deposition method has certain risks, and it is accompanied by some disadvantages, such as the preparation process is not economical, and the products are not pure.

Li et al. prepared large area graphene films by combining copper foil with graphene films in this way (Li et al., 2009). Remarkably, they found that the resulting graphene films have different effects on different thicknesses of copper foil, showing different graphene thicknesses, but no monolayers. In addition, Eda et al. successfully prepared molybdenum disulphide with good photoluminescence by means of hexane solution of butyl lithium (Eda et al., 2011). Due to the difference between 2D material structure and block structure, the band gap structure of MoS_2_ changed from indirect band gap to direct band gap after stripping from block material into layered material, resulting in the sudden increase of photoluminescence. This result provides a new direction for the structure research of nanoscale electronic materials. It is worth pointing out that Gao et al. report an ambient pressure CVD method to grow large-size, highly crystalline 2D TiS_2_ nanosheets through *in-situ* generating titanium chloride as the gaseous precursor, solving the high oxophilicity of active Ti precursors (Figures 1e,f). The addition of the NH_4_Cl promoter can react with Ti powders and switch the solid-phase sulfurization reaction into a CVD process, thus enabling the controllability over the size, shape, and thickness of the TiS_2_ nanosheets via tuning the synthesis conditions (Gao et al., 2018). In Figure 1g, high quality 2D metal-semiconductor NiTe_2_/MoS_2_ heterostructure is prepared by two-step CVD growth. Moreover, Zhai et al. enhance electronic behavior and optoelectronic response by the epitaxial growth of metallic vdW layered materials that can bring a new method to improve the performance of optoelectronic devices (Zhai et al., 2020).

### Liquid-Phase Exfoliation and Others

Liquid-phase exfoliation is also a common method. 2D materials that have been dispersed in the liquid phase are often expanded between their bulk layers, resulting in the reduction of intermolecular forces to the point where the ultrasonic wave produces enough energy to disperse the layers. As early as 2008, Coleman dispersed graphene in n-methyl pyrrolidone (NMP) solvent, used ultrasonic treatment for separation and centrifugation to remove impurities, and successfully prepared monolayer and less layer graphene without added oxides and structural defects through experimental tests (Coleman, 2009). Afterwards, to address the disadvantages of the above method, which requires a high boiling point and a large number of toxic basic solvents, they proposed another method: using surfactants to prepare graphene whose thickness is about 3 to 100 nm. Phosphorus can also be prepared in this way with the aid of the n-methyl-pyrrolidone (NMP) solvent (Brent et al., 2014). This method is also successful in the extraction of other 2D materials, with Smith et al. successfully preparing a variety of inorganic layered materials such as h-BN, dispersion forms of transition metal disulfides and transition metal oxides (Smith et al., 2011). For the summary study, Coleman summarized the methods of a large number of 2D materials in the liquid phase stripping technology, and discussed the spatial structure, application direction and mass production of various 2D materials (Coleman, 2009). In addition to the above-mentioned aspects, ion embedding and ion exchange methods in the liquid phase have also been studied in detail, providing a more intuitive direction for the future 2D material research.

Compared with the methods of Redox and CVD, the liquid-phase exfoliation method is relatively simple and can be applied to the industrial preparation of some 2D materials. However, the thickness of the material obtained by ultrasonic stripping is often uneven and the impurities are difficult to remove, which is also an important problem for ultrasonic stripping of 2D materials. Li et al. demonstrate that high-quality few-layer B sheets can be prepared in large quantities by sonication-assisted liquid-phase exfoliation, as shown in Figure 1h. By simply varying the exfoliating solvent types and centrifugation speeds, the lateral size and thickness of the exfoliated B sheets (Figure 1i) can be controllably tuned (Li et al., 2018).

Because some 2D materials, such as graphene, TMD and h-BN, are more hydrophobic than bulk materials, in most cases, the addition of biomolecules as a stabilizer is conducive to the successful stripping and can improve the preparation efficiency. For example, Laaksonen et al. make use of the hydrophilicity of bovine serum protein and obtain better results in the traditional liquid-phase exfoliation experiment for 2D material (Laaksonen et al., 2010). Taking graphene, the most typical 2D material, as an example, Raccichini et al. comprehensively evaluated several aspects of the production methods of several 2D materials that are often considered by researchers (Raccichini et al., 2014).

## Photocatalytic Properties of 2D Materials

For years, people have realized the seriousness of the energy crisis and environmental pollution problem, and the solar energy induced the decomposition of water molecules to produce hydrogen and the degradation of pollutants is one of the typical solutions to these problems. The application of traditional materials in the catalytic reaction is accompanied by some difficulties, such as difficulty in controlling the reaction efficiency and high price (Xie et al., 2013; Benck et al., 2014; Zhao et al., 2020). 2D layered materials have only a single or a few nanometer layers and large specific surface area which exposed more atomic and functional surface structures, providing a large number of active sites and more intense reactions. The introduction of vacancy, dislocation, impurity, and functional group in 2D materials will change the catalytic properties of materials and make 2D materials become new catalysts. Photocatalysis of 2D materials can convert light energy into chemical energy (carbon-based fuel), effectively alleviate the greenhouse effect and improve the utilization efficiency of solar energy. In the current research background, although the reaction efficiency and reaction speed of 2D materials for catalysis are still not widely used, the application and research progress of new materials are still of great significance.

**Figure 2:**
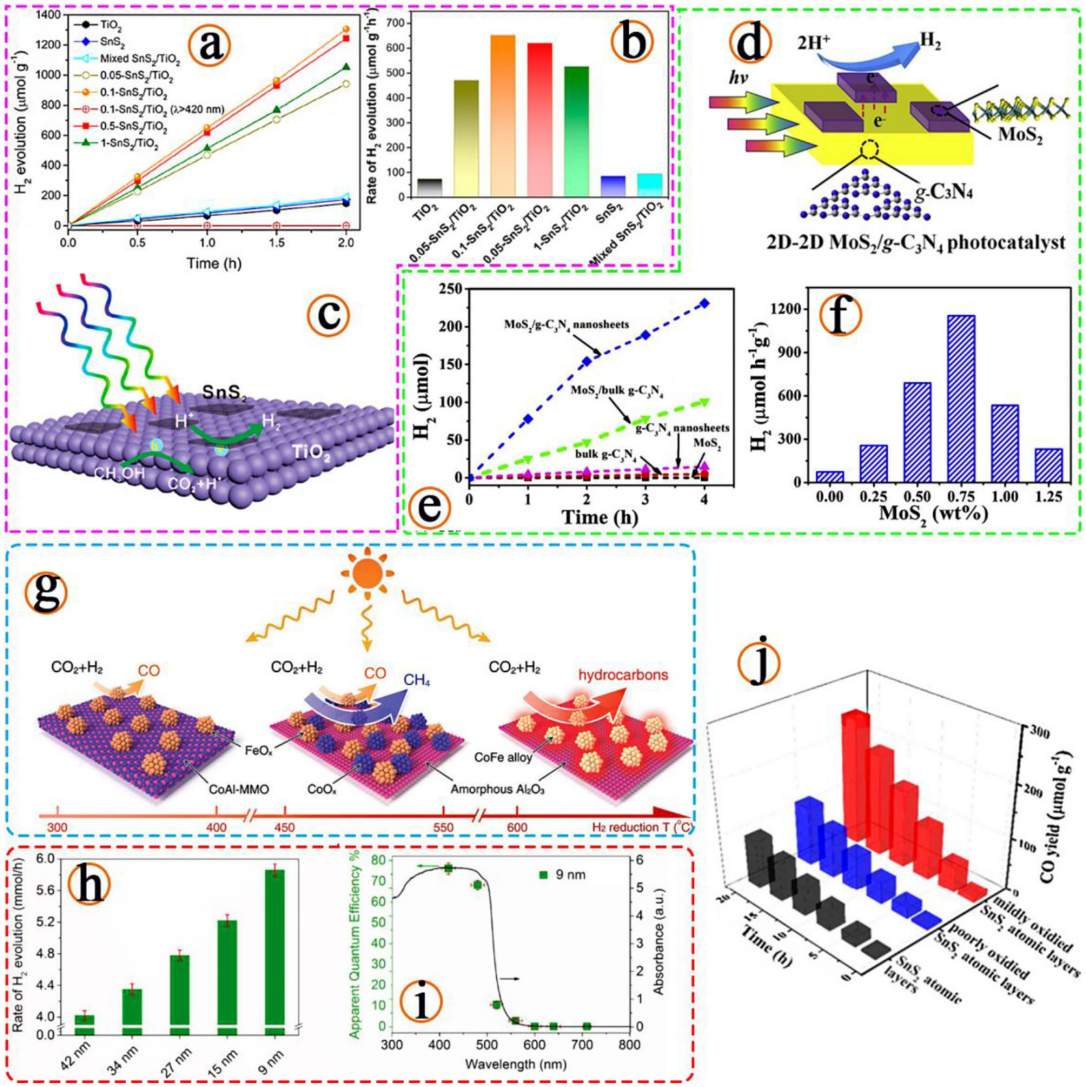
**(a)** Photocatalytic H_2_ evolution curves with different catalysts and **(b)** corresponding H_2_ evolution rates. **(c)** Schematic illustration of the photoinduced electron transfer process at the interface of the 2D-2D SnS_2_/TiO_2_ photocatalyst for hydrogen production, (Sun et al., 2019). Copyright 2019, American Chemical Society. **(d)** Schematic diagrams of 2D-2D MoS_2_/g-C_3_N_4_ nanosheets photocatalysts. **(e)** Time course of H_2_ evolution over different g-C_3_N_4_-based photocatalysts. **(f)** The effect of MoS_2_ amount on the photocatalytic H_2_ evolution performance, (Yuan et al., 2019). Copyright 2019, Elsevier. **(g)** Illustration of the different CoFe-x catalysts formed by hydrogen reduction of a CoFeAl-LDH nanosheet precursor at different temperatures and the CO_2_ hydrogenation selectivity of each CoFe-x catalyst is indicated, (Chen et al., 2017). Copyright 2017, Wiley. **(h)** The H_2_ evolution rates of MoS_2_/CdS composites with different lateral sizes of MoS_2_. **(i)** Wavelength-dependent apparent quantum efficiency of H_2_ evolution from the MoS_2_ (≈9 nm) loaded CdS composite, (Yin et al., 2018). Copyright 2018, Wiley. **(j)** Stability photoreduction test for 1, 4, 8, 12, 16, and 20 h of CO_2_ into CO under visible light irradiation for different samples, (Jiao et al., 2017). Copyright 2017, American Chemical Society.

As early as 1972, researchers pointed out that based on the transparency of water, the light wavelength reflected by photocatalytic water splitting should be <190 nm (Fujishima and Honda, 1972). Subsequently, more semiconductor materials that can be used as catalysts were reported, such as TiO_2_, ZnO, CdS, Fe_2_O_3_, SnO_2_, WO_3_ etc. However, traditional photocatalytic materials have some problems in the catalytic process, such as being uneconomic and having a large fluctuation of service life and low efficiency. Although conventional photocatalytic water splitting semiconductor materials have been developed for many years, their efficiency is still not up to the needs of industrialization. In the experiment of photoelectrode prepared by Liu et al. using heterogeneous WO_3_ and Mn group hydrogen evolution catalyst, they believed that the photoelectrode should meet some requirements such as wide range absorption, high carrier mobility, long carrier life, high stability, and environmental friendliness (Liu et al., 2010). In order to satisfy the above conditions, the preparation of effective photocatalytic water splitting catalysts by reducing the dimension of materials has attracted much attention from the research field. In the study of 2D semiconductor materials, these three directions are all conducive to improving the efficiency of semiconductor induced light reaction of 2D materials, extending the wavelength of the excitation reaction, reducing the recombination between carriers and increasing the active sites around the surface (Dong et al., 2019; Sun et al., 2019; Yuan et al., 2019), as shown in Figures 2a–f.

In the application of traditional catalysts, there is a method to control the catalyst activity by treating the induced material itself. A quintessential method is the manufacture of TiO_2_ (Chen J. et al., 2020) doped composite semiconductor with good catalytic effect, including but not limited to SnO_2_/TiO_2_ (Kusior et al., 2018), WO_3_/TiO_2_ (Wang et al., 2019), MoO_3_/TiO_2_ (Liu et al., 2016), SiO_2_/TiO_2_ (Cui et al., 2019), and ZrO_2_/TiO_2_. The development of 2D material catalysts is similar to that of traditional materials. Some people have found that the strong catalytic activity of 2D materials on composite nanocomposites based on density functional theory is due to the narrowing of band gaps. It is also demonstrated that 2D materials can be combined with effective photocatalysts to improve efficiency (Yang, 2017). The structures and properties of various nanocomposites are also studied comprehensively by density functional theory, and the theoretical results are verified by experiments (Zhao et al., 2021). Figure 2g shows that the change of reaction temperature and time used to prepare the material will affect the morphology of the material, different reaction conditions also have a certain degree of influence on the catalytic performance (Chen et al., 2017). Meanwhile, the above study laid a foundation for the photocatalytic application of 2D composites.

### Photocatalytic Activity of Graphene

Graphene, one of the most commonly used 2D materials, is also used in catalyst applications. As summarized by Deng et al. although the effect of the original graphene in catalytic reactions is often inert, the change in the electronic structure of graphene controlled by the crystal mechanism leads to the increase of the catalytic activity of graphene (Deng et al., 2016). They classified these methods into five types: size effect, layer effect, edge and defect effect, curvature effect, dopant, and functional group effect.

Firstly, from the point of view of the photocatalytic water splitting of 2D materials to produce hydrogen, Xiang studied the composite photocatalyst (graphene/g-C_3_N_4_), which was prepared by calcining and impregnation reduction after the reduction of flowing nitrogen and hydrazine hydrate at 550°C (Xiang et al., 2011). It has been found that when graphene is used as the basis, the structure of graphene will affect the structure and optical properties of g-C_3_N_4_ catalyst, because graphene can effectively separate photogenerated charges as a conductive channel, thus improving the photocatalytic activity of g-C_3_N_4_. After a large number of experiments, the researchers compared the data and found that the best catalytic effect was when the mass content of graphene was 1.0%, the corresponding hydrogen production was 451μmol·h^−1^·g^−1^, 3.07 times higher than that of pure g-C_3_N_4_. What is more, Xiang et al. concluded that graphene is a promising dopant material in the visible light wavelength range and can be used to develop high-performance optical catalysts, which can also be used in sewage treatment and dye-sensitized solar cells. Similarly, Reduced GO (RGO) is seen as a carrier, a semiconductor, and a metal nanoparticle. Photoelectrons from the semiconductor can be transferred via RGO after exposure to ultraviolet light. This ability allows it to be used as a nano-catalyst material, since electrons can be transferred to the adsorbed material. Along with the investigation, the study of these composites can promote the industrialization of photoinduced hydrogen evolution reaction (Lightcap et al., 2010).

Photocatalytic degradation refers to the process of degradation of pollutants into innocuous outcome through the reaction process between free radicals and organic pollutants by using the highly active free radicals and photocatalysts produced by radiation in the reaction system. Wang et al. synthesized a new composite material, a graphene-based ultrathin barium titanate nanosheet with exposed {001} flat surface, with good photocatalytic activity (Wang et al., 2012). Through the success of the development of the new material, they summarized several key factors affecting the catalytic performance through spectral analysis, and gave several examples, including the formation of Ti-O-C bonds leading to a wider light absorption range, better charge separation rate, and a larger catalytic surface.

Graphene has the ability to adsorb pollutants and act as a semiconductor photocatalyst to degrade dye wastewater. The photocatalytic materials after 2D composites can not only rapidly transfer photogenerated electrons and rapidly separate electron holes, but also improve the adsorption performance of dyes. This kind of recombination 2D material has a broad application prospect and research value in the field of environment, because of its adsorption and catalytic degradation of environmental pollutants, such as heavy metals, dyes and persistent organic compounds, as well as environmental detection (Shen et al., 2014). Fan et al. compared the photocatalytic degradation performance of ZnO/GR composite nanosheets with that of ZnO and other materials by hydrothermal reaction and found that the catalytic performance of the composites was better (Fan et al., 2012). They believe that the photolithography of ZnO is inhibited by the composite material and ZnO/GR will be one of the key candidate materials for new catalysts. Doping of 2D composites is also a common method to improve their properties. One example is that Farhangi et al. doped titanium dioxide composite graphene sheets with iron and passed the sol-gel method (Farhangi et al., 2014). Compared with pure materials, the new Fe-TiO_2_ with functionalized graphene flakes has better visible light absorption and conversion efficiency and higher photocatalytic activity. This experiment brings us to the conclusion that the photocatalytic intensity can be changed by improving the absorbability of dyes, expanding the optical response range, and improving the charge separation and differential transport performance. In addition, they carried out density functional calculations and found that the doping of atomic points works best when they are in the middle, which experimental results proved. This study of degradation provides a new way of thinking and direction for the study of 2D composites, which combines theory with practice.

### Photocatalytic Activity of Transitional Metal Sulfides (TMDs)

In addition to widely studied Graphene, MoS_2_ is also a common semiconductor catalytic material and is often used in 2D material induced photocatalytic experiments. In 2016, in the experiment of the photocatalytic performance of monolayer molybdenum disulfide on nanocomposites studied by Ding et al. the reasons for improving its photocatalytic activity and stability were explored, and the role of monolayer molybdenum disulfide as sensitizer and auxiliary catalyst (Ding et al., 2016). Compared with pure SnO_2_, the bandgap of composite materials is smaller (0.49 eV), and the optical absorption spectrum can completely cover the visible region, even in the infrared region. They suggest that this dual effect may also be present in other MoS_2_ semiconductor nanocomposites, paving the way for researchers to develop highly effective MoS_2_ hybrid photocatalysts.

In the study of visible light catalytic carbon dioxide reduction reaction, 2D SnS_2_ with partial oxidation state has good catalytic activity, because the material structure promotes the separation of electron holes, changes the adsorption energy of molecules, and improves the photocatalytic efficiency (Jiao et al., 2017; Figure 2j). In the experimental results, surface photovoltage and photoluminescence spectroscopy confirmed that the presence of hybrid oxides is conducive to the separation of charge and carrier. *In situ* Fourier transform infrared spectroscopy fully indicated that the main intermediate product of CO_2_ photocatalytic reduction is COOH^*^. In addition, the researchers calculated by density functional theory that with the help of stable COOH^*^ intermediates, the electron positions on Sn atoms generated by local oxidation can reduce the activation energy barrier of CO_2_. Due to the above characteristics, the oxidized SnS_2_ atomic layer showed a carbon monoxide production rate of 12.28 in the molg^−1^h^−1^, which was higher than the slightly oxidized and unoxidized SnS_2_ by 2.3 and 2.6 times. In conclusion, it can be proved experimentally and theoretically that locally oxidized regions can improve the efficiency of charge separation and promote the activation of carbon dioxide and open up new ways to reduce the greenhouse effect.

Similarly, by contacting the edges to create more active catalytic sites, monolayer molybdenum disulphide is synthesized with rich active sites by gradient liquid-phase exfoliation (Yin et al., 2018), as shown in Figures 2h,i. The prepared molybdenum disulphide shows excellent performance in the generation of H_2_ by photocatalysis. The optimized monolayer MoS_2_ nanometer sheet with an average transverse size of 9 nm can significantly improve the photocatalytic performance of MoS_2_ nanometer sheet through synergistic action and lateral size change due to the increase of the active site density and the shortening of the charge diffusion distance.

### Photocatalytic Activity of Graphitic Carbon Nitride

Graphitic carbon nitride (g-C_3_N_4_) with high physicochemical stability, a narrow band gap (2.7 eV), tunable electronic structure, low cost, non-toxicity, and most importantly, appropriate band edge, as a typical two-dimensional (2D) metal-free conjugated polymer semiconductor material, has been broadly applied in photocatalysis for H_2_ production and CO_2_ photoreduction. However, the limitations of g-C_3_N_4_, such as poor electric conductivity, trivial charge flexibility and disadvantaged visible light utilization, have been found in the study, which greatly limited its performance in photocatalysis. In that case, a variety of methods have been proposed to improve catalytic performance, including textural property design and constructing semiconductor heterojunctions, etc. Metal sulfides have attracted wide attention in photocatalytic hydrogen production because of their suitable band gap and catalytic function. SnS_2_ was supported on 2D g-C_3_N_4_ for excellent and stable visible light photocatalytic hydrogen generation (Jing et al., 2018). The SnS_2_ nanoparticles/g-C_3_N_4_ composites improving the rate of electron-hole pair separation exhibit the highest visible-light-driven H_2_-generation rate of 6305.18 μmol h^−1^ g^−1^ without any noble metal as cocatalyst. And a mesoporous 3D/2D NiCoP/g-C_3_N_4_ heterostructure was synthesized (Li et al., 2020). This work has indicated that multidimensional heterojunctions of transition metal phosphides supported on g-C_3_N_4_ can dramatically increase the photocatalytic activity and stability by fabricating surface metal-N bonding states. These findings regarding the design, fabrication and photophysical properties of heterostructure systems may find use in other photocatalytic applications including CO_2_ reduction and water purification. g-C_3_N_4_/NiAl-LDH 2D/2D hybrid heterojunction is used for high-performance photocatalytic reduction of CO_2_ into renewable fuels (Tonda et al., 2018). In the process of photocatalysis, the construction of heterojunctions by combining two different semiconductors, especially the two-dimensional (2D) layered structure with appropriate conductive potential and valence band potential, is one of the most effective ways to improve the separation efficiency of photogenerated carriers. In addition, two-dimensional (2D) g-C_3_N_4_/BiOCl heterostructures demonstrate a facile strategy of interfacial oxygen vacancies (IOVs) with enhanced interfacial interaction to promote the photocatalytic conversion of CO_2_ (Chen Y. et al., 2020). Z-Scheme assembly of 2D ZnV_2_O_6_/RGO/g-C_3_N_4_ nanosheets with RGO/pCN as solid-state electron mediators enables efficient CO_2_ conversion under visible-light irradiation (Bafaqeer et al., 2019).

As mentioned above, in the study of catalytic performance of a variety of 2D materials, the introduction of functional 2D materials into a variety of semiconductor photocatalysts, or other methods such as adding semiconductor photocatalysts with 2D materials as the matrix can enhance the photocatalytic reactivity by virtue of the unique properties of 2D materials. These physical or chemical bonds made of 2D materials enable the composite to possess unique properties, such as multiple active sites, increased optical response range, and enhanced charge separation and transport performance, thus enhancing the photocatalytic performance of 2D materials. This has a good effect on our understanding of the photocatalytic reaction mechanism and the creation of industrializable catalysts.

2D materials have great structural advantages in the field of photocatalysis because of their high electrochemical nature on both basal planes and edges of 2D materials in energy conversion (e.g., TMDs), well-balanced hydrogen binding Gibbs free energy on the basal sites (e.g., 1T′ MoS_2_), high specific surface area and abundant surface active sites, and the external performance is that 2D materials have outstanding catalytic performance. Not only the excellent performance, but also the unique 2D structure, is fairly attractive to researchers, providing advantages such as such as high cooperation. On this basis, the synthesized heterostructure and regular composite structure have better performance and work efficiency in the process of research.

## Conclusion and Outlook

Regarding the preparation of 2D materials, we mainly talked about several preparation processes, such as the mechanical exfoliation method, Redox method, chemical vapor deposition method and ultrasonic liquid exfoliation method. In the preparation of materials, we pay special attention to the structure of the materials, because the catalytic effect of 2D materials is often related to morphology, phase structure, and porosity. For instance, pure graphene is inert, but after doping, it has a good catalytic performance. MoS_2_ and WS_2_ nanosheets not only have the effect of photolysis themselves, but also can replace precious metals as composite catalysts.

Generally, there are pure 2D catalysts which will utilize the unique characteristics of 2D materials in the catalytic reaction to improve the catalytic efficiency, 2D materials as doping or as carriers and other methods to prepare catalysts. Representative is the combination of 2D materials with traditional precious metal catalyst of Pt. At the same time, many processes to enhance the catalytic effect are introduced, including enhancing the transmission of charge, increasing the surface active sites, and lengthening the absorption spectrum. Obviously, part of the above preparation methods are first calculated by density functional principle and then verified by experiments and that also explains the importance of studying the catalytic performance mechanism of 2D materials. In the reports of these catalysts, a slice of them have their own styles and unique preparation methods, which have opened up new ways for researchers to improve performance, enhance stability and improve yield.

It is widely believed that 2D materials have good catalytic potential. Although many good 2D materials or 2D materials based catalysts have been introduced above, the application of 2D materials in catalyst research is not limited to that and the research and development of new hybrid 2D material components still have great prospects. We believe that under more research background, under more scientific and theoretical analysis of 2D material structure, 2D catalysts will be more satisfactory in terms of preparation, efficiency, and stability. Even applying it to the process of industrialization does not take much time. In the future, the structure and morphology of 2D materials will be more diversified.

In addition, 2D structures are used as the foundation to participate in the design of more superstructures, including heterostructures, metal and non-metal doping, and coating. Even more prominent properties of 2D structures, such as magic horn graphene, will be discovered. And the realization of these prospects may require the efforts of researchers and the study of the problem from a different perspective.

## Author Contributions

All the authors contributed in preparation, characterization and analysis structure, performance of materials, discussed the results, and commented on the manuscript.

## Conflict of Interest

The authors declare that the research was conducted in the absence of any commercial or financial relationships that could be construed as a potential conflict of interest.
